# How do I deal with breast cancer: a qualitative inquiry into the coping strategies of Iranian women survivors

**DOI:** 10.1186/s12905-022-01865-0

**Published:** 2022-07-08

**Authors:** E. Manouchehri, A. Taghipour, A. Ebadi, F. Homaei Shandiz, R. Latifnejad Roudsarii

**Affiliations:** 1 Department of Midwifery, Faculty of Nursing and Midwifery, Mashhad Medical Sciences, Islamic Azad University, Mashhad, Islamic Republic of Iran; 2 Department of Midwifery, School of Nursing and Midwifery, Mashhad University of Medical Sciences, Mashhad, Islamic Republic of Iran; 3 Department of Epidemiology, School of Public Health, Mashhad University of Medical Sciences, Mashhad, Islamic Republic of Iran; 4 Social Determinants of Health Research Center, Mashhad University of Medical Sciences, Mashhad, Islamic Republic of Iran; 5 Behavioral Sciences Research Center, Lifestyle Institute, Baqiyatallah University of Medical Sciences, Tehran, Islamic Republic of Iran; 6 Nursing Faculty, Baqiyatallah University of Medical Sciences, Tehran, Islamic Republic of Iran; 7 Cancer Research Center, Mashhad University of Medical Sciences, Mashhad, Islamic Republic of Iran; 8 Nursing and Midwifery Care Research Center, Mashhad University of Medical Sciences, Mashhad, Islamic Republic of Iran

**Keywords:** Breast cancer, Content analysis, Coping strategies, Coping styles, Iranian women

## Abstract

**Background:**

Breast cancer is the most frequent cancer in Iran. Understanding the coping strategies employed by cancer survivors can provide valuable information for designing interventions to help them adapt to the problems produced by cancer and its treatment. This study aimed to explore the coping strategies of BC survivors in Iran.

**Methods:**

This qualitative study was conducted in Mashhad, Northeast Iran, between April and December 2021. Fourteen BC survivors were selected through purposive sampling. The data were collected using semi-structured interviews. Data were analyzed using conventional content analysis adopted by Graneheim and Lundman. MAXQDA 12 software was used for data organization. Components of trustworthiness, including credibility, dependability, confirmability, and transferability, were considered.

**Results:**

The main categories that emerged from the participants' data analysis were “behavioral coping strategies” and “emotional coping strategies.” Behavioral coping strategies included efforts to adopt healthy nutrition, attempts to improve a healthy lifestyle, maintenance of everyday activities, use of specialized cancer support consultation services, and seeking to increase health literacy about BC. The emotional coping strategies consisted of denial as a temporary escape route, positive thinking and focusing on the positive aspects of life, reinforcement of spirituality, and seeking the support of relatives.

**Conclusion:**

Our findings provide an in-depth understanding of Iranian women’s strategies for coping with BC. A trained team of oncologists, psychiatrists, mental health professionals, and reproductive health specialists needs to contribute significantly to improving the coping ability of patients with cancer, which could lead to enhanced health promotion and a higher quality of life.

## Introduction

Cancer is a complex disease that affects patients in both physical and emotional aspects. The diagnosis of breast cancer (BC), followed by medical treatment and additional life challenges, can be associated with significant emotional distress [1, 2]. According to the results of several studies in Iran, losing the breast for a woman is not just the loss of a part of the body but also affects a person's feelings towards others and their reactions, as well as their lifestyle, quality of life, perceived threat to continuing marriage bound, and even if it is followed by social complications [3–5].

In Iran, the breast is perceived as one of the most private parts of the body and a significant part of a woman’s identity, serving as a symbol of femininity, sexual desire, beauty, and a woman’s capacity for motherhood [3, 4]. Also, talking about it is considered taboo. Some women, despite seeing suspicious symptoms in their breasts, see a doctor too late and are reluctant to have a clinical breast examination, especially by a male therapist [8–10].

Worldwide, BC survivors have some sequelae, which result in negative feelings [5]. The sequelae may include hair loss, infertility, weight gain, or premature ovarian failure and sometimes limited ability to move an affected arm, changes in body image, dyspareunia, reduced sexual desire and lower sexual attraction for the partner, and therefore, low self-esteem [5–9].

Some authors have posed coping as one of the factors that can determine wellbeing [10, 11]. Coping strategies, as one of the factors that influence wellbeing, are the cognitive and behavioral efforts of individuals to interpret and overcome problems that generally include three types problem-focused, emotion-focused, and avoidant coping [12].

The goal of coping strategies is to compensate for or improve stressful situations by either the reformulation of objectives or the adjustment to a new and positively assessed situation [13]. For women with BC, coping is a strategy through which they perceive and handle various stressors experienced during the BC diagnosis and treatment process as challenges and threats. Such coping strategies influence psychosocial adjustment among BC survivors [14].

Coping with the disease is a dynamic process that is strongly influenced by the individual and cultural factors [15] and may take different forms between and within cultural and religious traditions [16]. It has been reported that the choice of coping strategies is designed by national culture [17, 18].

Due to the prevalence of BC in Iran and its occurrence at a younger age than in western countries [19], several qualitative studies have been conducted to investigate the problems and issues of women with BC in Iran. Some of these studies have been conducted in many years ago or carried out in a limited time period after the diagnosis and treatment of BC [20]. Some other studies have been conducted with different qualitative methods [21, 22], either on the positive aspects of life changes [23] or spiritual aspects [24] or facing the feeling of pity [25], or receiving support [25, 26] and finally, the problems related to childbearing age in BC survivors [27]. Therefore, given the rapid and significant advances in BC diagnosis and treatment methods in recent years, as well as considering the particular cultural context of Iran, a qualitative study that explores all aspects of BC coping strategies in Iranian women seems necessary.

Such studies can provide valuable information for designing interventions to help cancer survivors to cope with the problems caused by disease and its treatment [28]. So, this study aimed to explore the coping strategies of BC survivors in Iran using a qualitative approach.

## Materials and methods

### Study design

We used a qualitative descriptive study to explore BC survivors’ coping strategies in Iranian women. This method is suitable for subjects focused on “what” questions about human interpretations and opinions. Real-life is different for each person, and this method tries to describe the phenomenon (coping strategy) in the best possible way. It also tries to interpret the findings without going too far from that literal description [29].

### Participant selection

Participants were recruited for the study through purposive sampling [30] in various clinical settings in Mashhad, one of the leading referral cities in the east of Iran. In order to provide maximum diversity, participants were selected from both governmental and private clinics. The participants received a diagnosis at least 6 months prior to the interview. Some of the participants underwent chemotherapy, and some of them were women who had completed their treatment and attended follow-up visits. Women who had incurable stages of BC, metastatic diseases, and concomitant other malignancies were not interviewed.

### Data collection

Data was collected through in-depth, semi-structured, and face-to-face interviews using an interview schedule provided based on the development strategy of a semi-structured interview guide, including five phases of: (1) Identifying the need for using semi-structured interviews (2) Retrieving and applying prior knowledge (3) Formulating the preliminary semi-structured interview guide (4) Pilot testing the guide and (5) Presenting the complete semi-structured interview guide [31]. Concerning our research topics, the semi-structured interview method was suitable for studying BC survivors’ perceptions and experiencing regarding coping strategies. Also, in a semi-structured interview, it was possible to focus on the meaningful issues to the participant, allowing diverse perceptions to be expressed. With respect to applying prior knowledge, we reviewed previous literature to create a predetermined framework for the interview. Furthermore, we consulted with the three members of the research team (RLR, AT, and AE) who are skilled in qualitative studies for methodological guidance and feedback. To formulate the preliminary semi-structured interview guide the interview guide questions were designed to achieve the richest possible data. The questions were not leading and also clearly worded, single-faceted and open-ended. They consisted of two levels of questions: main themes and follow-up questions. In this way, our interview guide could produce data, allowing new concepts to emerge. For pilot testing, we used field testing. In this regard, the interview guide was piloted with three participants. The interviews were conducted by the first author (EM), who has a background in health research and clinical obstetrics and gynecology. Questions were about participants copying strategies and disease management styles for BC included: “Please explain your feelings when you found out that you have got BC?/”In your opinion, what conditions in your life could reduce your BC recurrence?/How did you cope with the diagnosis of BC?” Exploratory questions such as “Can you explain more?” or “What do you mean by this sentence?” were also used to provide further explanations. Interviews lasted approximately 70–90 min.

Interviews were continued until data saturation when no new code emerged from the interviews [32, 33]. The gold standard method of determining the purposive sample size in health science research is data saturation which refers to the time when no new code is obtained in data coding [34, 35]. They were conducted and analyzed in the Persian language. Subsequently, only the passages used as quotes were translated into English. It is noteworthy that the quotations from non-English interviews could be utilized if the researchers have further awareness of sensibility toward dealing with language and translation issues [36]. For this reason, quotations were translated by a bilingual expert who was fluent in both the Persian and English languages. The interviews were recorded and immediately transcribed verbatim. Data was collected from April to December 2021.

### Data analysis

Content analysis was used to explore and classify the self-reported coping strategies of participants as emerging from the original interviews adopted by Graneheim and Lundman. This method includes two steps of: de-contextualization (selecting and condensing meaning units, and coding), and re-contextualization (creating categories on various levels) [37]. Transcripts of the initial three interviews were reviewed by the authors (EM, AT, AE, and RLR) to establish a preliminary coding template used for subsequent analysis. All transcripts were then analyzed. Codes were generated from the interview data and systematically applied to identify subcategories and categories.

The process was iterative, reflexive, and interactive as continual data collection and analysis shaped each other. For example, code titles or descriptions based on earlier interviews were revised according to the data collected during subsequent interviews. Once all transcripts were initially coded, the team reviewed the coding and elicited discussion about the coding strategy, attempting to achieve consensus to resolve coding discrepancies and merge individual codes into overarching categories. Data organization was carried out by MAXQDA 12 software. MAXQDA provides a variety of code- tools, permitting an explicit, suitable and easy coding effort and supports data organization in qualitative research [38].

### Trustworthiness

To confirm the validity and accuracy of this qualitative research, four criteria presented by Lincoln and Guba, including credibility, dependability, confirmability, and transferability, were examined [33, 36].

In order to maintain credibility, the researcher tried to recruit and interview informant participants to collect rich and in-depth data regarding the concept under study. Also, the long-lasting engagement of the researcher in the field with participants allowed for building trust and obtaining rich data, which enhanced the rigor of the study. Also, the researcher's interest in the study subject, constant involvement with data, review of audit and qualitative research experts, and search for evidence and other articles are indispensable. To guarantee dependability, an external audit (both the process and product of the research study) was done by two expert researchers not involved in the research process. In addition, the authors discussed the coding and analyses throughout the research process and reached an agreement with the final coding framework and themes, which made both data credibility and dependability possible [39]. To ensure confirmability, several qualitative researchers were also consulted, and the researcher tried to describe the method of study in detail. To increase the transferability, purposive sampling was used, and interviews were conducted with different participants with maximum diversity, and direct quotations and examples were provided. Also, it was tried to enable the transferability of study results to similar populations through a detailed context description [40].

### Ethical considerations

Before the beginning of each interview, the objectives of the research, the reason for recording the interview, voluntary participation, confidentiality of the information, and the interviewer’s identity were explained, and written consent was obtained. All information collected from participants was kept confidential and anonymous, i.e., instead of using their names, women were given codes to be used in the analysis. At the end of the interview, a gift was given to the participants for compensation and to express of appreciation.

## Results

Participant's demographics: The characteristics of participants are shown in Table 1. All of the participants were Muslim.

**Table 1:** Description of participants (n = 14)

Participants characteristics	No. (%)
*Age*	
30–39	4 (29)
40–49	4 (29)
50–59	6 (42)
*Literacy*	
Primary	4 (29)
Diploma/secondary	3 (21)
University	7 (50)
*Occupation*	
Housewife	7 (50)
Employed	7 (50)
*Marital status*	
Married	9 (64)
Divorced	3 (21)
Single	2 (15)
*Types of cancer clinic*	
Public	7 (50)
Private	7 (50)
*Status of treatment*	
Under chemotherapy	8 (57)
Completed treatment	6 (43)
*Total mastectomy*	
Yes	9 (64)
No (breast-conserving surgery)	5 (36)

The data saturation was achieved after 14 individual semi-structured interviews with participants. Overall, 492 codes were extracted, and the codes related to the research objectives were finally abstracted into eight subcategories and two categories (Table 2).

**Table 2:** Categories, and subcategories emerged from the data analysis

Sub category	Category
Efforts to adopt healthy nutrition	Behavioral coping strategies
Attempts to improve the lifestyle	
Maintenance of everyday activities	
Use of specialized cancer support consultation services	
Seeking to increase health literacy	
Denial as a temporary escape route	Emotional coping strategies
Positive thinking and focusing on the positive aspects of life	
Reinforcement of spirituality	
Seeking the support of relatives	

Women engaged in numerous types of coping strategies to deal with BC. The two main categories within coping were behavioral and emotional coping strategies.

### Category 1: behavioral coping strategies

Women engaged in several coping strategies that involved behavioral changes or maintenance of behavior, emphasizing on prophylactic for reducing the risk of disease recurrence, seeking information, and self-care behaviors. This category includes efforts to adopt healthy nutrition, attempts to improve the lifestyle, maintenance of everyday activities, use of specialized cancer support consultation services, and seeking to increase health literacy about BC.

### 1a-Efforts to adopt healthy nutrition

Most participants cited changing their diet to adapt to BC by switching to a healthier diet and using higher quality foods: “I use yellow beef oil myself. It works. As soon as I eat Indian rice and liquid oils, my body reacts. I feel very terrific when I use natural products. In the mornings, I mix oatmeal, wheat germ, almonds, honey, and water and eat it. I have got tremendous energy. I also use a lot of fruits and vegetables … I said God, and his miracle is undeniable. His miracle is in fruits and plants. My body comes back. I still believe the same thing…” (p8).

### 1b-Attempts to improve the lifestyle

Almost all participants mentioned lifestyle improvement as one of their ways to cope with BC. One university teacher, one of the participants, said: “I tried to reduce my stress. I tried to lose weight, but I failed. I take drugs for effective prevention (of BC). This is what I did. However, I need to lose more weight. Obesity is a risk factor. I tried to make my diet healthier and thought about our diet. I must not use everything.” (p10).

A housewife with high socio-economic status said: “To prevent it (BC), I try to reduce my stress. I try not to get too involved in the problems and not care too much about them. I have nothing to worry about right now. I go for a walk, do yoga, listen to music and …” (p7).

### 1c-Maintenance of everyday activities

Women reported that staying busy was a helpful coping mechanism. Participants stated that they try to do all the housework themselves, such as cooking, making pickles and jams, shopping, even going to a sewing class, walking, participating in public activities, attending their workplace, and doing their job responsibilities. In fact, by doing these activities, a sense of control over the current situation was created for them. Nearly all the participants reported that business would distract them from their anxiety or anger. Whether it was staying busy with shopping, traveling, or doing excessive activities at home. Women in our sample preferred to stay busy to keep their minds preoccupied: “I decided to be in the shop, doing clothes repairs, making clothes products, going out of the house. It was great for me, and it worked a lot. Because when I stay at home, I am greedy. I am perfect now. “ (p8).

Another participant stated, “I travel a lot. After my operation this year, I traveled five times. I travel regularly. I mostly go out of town. I spend time with my friends. I go mostly to parties.” (p11).

### 1d-Use of specialized cancer support consultation services

Some participants considered the importance of using supportive, counseling, and professional services. Access to such services reassures cancer survivors and reduces concerns about the disease and its treatments. However, in most cases, these services only cover patients' physical problems, and counseling on patients' psychological and emotional issues remains unspoken and unresolved. They expressed dissatisfaction with the lack of access or lack of such services and considered it a necessary need of their peers: “The problem is that wherever you go, they only talk to you about your physical problems. While someone like me needs encouragement and emotional support so that we can cope more easily with the disease.” (p2).

Nevertheless, a few participants were satisfied with receiving these services: “It is excellent here. They take care of us like their own family. They also give us other advice on how to eat and any problems we may have. They’ve developed a group in cyberspace and answered our questions at any time …” (p5).

### 1e-Seeking to increase health literacy about BC

Most of the participants mentioned that they used different sources of information about BC and the most appropriate and best ways to deal with it: “I have already found what would happen in the treatment process in the pages where several affected women are present.” (p12)

Another participant about her information source for BC said: “Gradually, I found it is a curable disease by researching the web and everywhere and studying and inquiring from those infected. Now the path may be difficult. But finally it is curable.” (p13).

However, one of the participants preferred not to learn much about the disease and its course because she thought getting more information would increase her stress and make her feel out of control. A 45-year-old woman with BC said: “Unlike some who are looking for information, I did not want to know the details of my illness. I felt that I might lose hope of recovery.” (p14).

Some participants mentioned their presence in support networks or peer groups as an appropriate source of experience and awareness transfer. The existence of such supportive groups encouraged women to discuss their problems and experiences openly and create a supportive atmosphere within which to exchange knowledge and awareness concerning the resources and facilities. This sort of interaction and mutual communication, as well as the formation of support groups and the exchange of successful experiences, was shown to be helpful for patients: “When I raise my issues and problems in these groups, I feel that someone is responding to me who has already experienced this problem and has chosen the best path for herself.**”** (p4).

### Category 2: Emotional coping strategies

Women also used “emotional coping,” which is a way to deal with stress and minimize the effects of BC on their lives and was often used before behavioral strategies. These strategies included denial as a temporary escape route, positive thinking and focusing on the positive aspects of life, reinforcement of spirituality, and seeking the support of relatives.

### 2a-Denial as a temporary escape route

In the interviews of many participants, their first reaction to BC was psychological denial: “I was distraught when I heard I had cancer. Even though I knew it was not very dangerous and had a better prognosis, it was still taboo for me. I could not believe it. Even the word “cancer” was too heavy for me. However, I knew it was better than other cancers.” (p10).

“I became very ill after hearing that I had BC. I mean, I never thought I was in this world. I thought no, this is not for me. It may be for everyone, but not for me.” (p11).

### 2b-Positive thinking and focusing on the positive aspects of life

Several women reported a sense of gratitude toward their experience with BC treatment and being BC survivors. Many reported feeling grateful because their BC had given them the chance to appreciate life, value their family, and feel close to God.

Many reported expressing positive emotions toward their families and reassuring them that they were fine, although they genuinely felt the opposite. Some participants were even reluctant to associate with relatives who caused them frustration: “Now, I go to different parties, including wedding parties. I will not even put on a wig. Because I say, this is fashion. Everyone says how beautiful it is. They encouraged me, and I did not feel any discomfort. I have no deficit, and I follow the same routine as before.”(p1).

Some participants even controlled their negative thinking about the disease: “I will not allow anyone to draw a black picture of my illness. This is my attempt. When I come for chemotherapy, I listen to music and read books. It is very effective to be able to control yourself. Do not let anyone disappoint you.” (p6).

“I think the most important factor for coping with any disease is the patient herself. That is, she must first want to accept the disease. Nothing could be done about what would happen. However, in the future, we can do preventive measures like annual screens and laboratory tests. Every time you test and see that everything is OK, you will be happy. Put yourself in a position to try to reduce the risk. To prevent metastasis, exercise and a quiet, stress-free lifestyle are what you can do to help yourself. Moreover, this must no longer be a big part of your life. I mean, I deal with this disease so that it is just something that happened to me, and now I want to live a life with it that is low on stress and high on peace and joy. Maybe if we observe all these things, this disease will gradually disappear from our lives. That means you forget that you have such a disease because you’ve accepted it. I did not let BC be the focus of my life, so, you know, it went to the sidelines.” (p10).

### 2c-Reinforcement of spirituality

All participants, regardless of their religious background, dealing with cancer as something coming from God that they had to accept because they had no power to change the situation. A 50-year-old divorced lady said: “I think everyone has a lifetime. Who am I in the world? I do not think about the recurrence of the disease and what to do with it. I do not have a husband. When my son and daughter-in-law leave, I plan to make wallpaper, paint, change my curtains, travel, and do other things… You know, we can’t anticipate the future. Everything is in the hands of God.” (p2).

The majority of women reported relying on their religiosity to help them cope. Women relied on religious practices, such as reading the Bible or praying, to help them when they experienced the onset of negative emotions: “When I realized that I had BC and had to have surgery, I then went to the holy shrine of Imam Reza and asked him to give me strength. I was crying a lot. I said, give me the strength to go to the operating room. I said, how difficult is this divine exam? However, I will come out of this exam with pride. I talked a lot there and recited the prayer, becoming stronger. Then I went to the operating room myself… You have to think it is God's will. God wanted us to be like this. God's destiny cannot be countered.” (p9).

### 2d-Seeking the support of relatives

Women reported that many relied on their families as a source of support. The majority of participants stated that they noticed their spouse’s depth of empathy and loyalty after their BC diagnosis, and some stated that their spouse’s behavior has changed and he has become kind and good-natured, unlike in the past. Women mentioned family as a source of fortitude, whether it was through a husband or daughter, neighbor or relative: “My mother-in-law is very careful of me, she comes here and cooks for me. It has had a great influence on me. She seems like a kind and compassionate friend. It makes me very happy.” (p2).

A woman experienced the support of her family through this process: “Thank to God, God gave me much patience. My husband was accommodating. My children got along very well with this problem, and my mother was very supportive too.” (p11).

Nevertheless, one of the participants did not receive such care from her relatives: “My friends and neighbors came to see me, talked to me, assured me, and prepared food, but my daughter-in-law did not do it for me. In these difficult circumstances, I realized that strangers are better than my family.” (p9).

Some participants viewed human behaviors towards them, accompanied by exaggerated affection from relatives or friends, as barriers to their coping with the disease and creating distressing feelings. They did not believe they deserved such sympathy and compassion but rather ordinary, non-exaggerated behavior. They believed inappropriate behavior would disturb their peace and worsen their condition. Their social isolation was triggered by their behavior towards people who identified them as having a dangerous disease: “In the early stages of the diagnosis of my illness, I went out to party several times. Nevertheless, I encountered behaviors that made me upset. Some bothered me with too much compassion and attention, and some with telling stories about cancer. Their words and behaviors made me feel worse. Now, I won’t go to the party at all. I feel more comfortable at home.” (p2).

## Discussion

The present qualitative study was performed to explore the coping strategies of Iranian women with BC. According to this study, women in Iran use emotional and behavioral strategies to deal with the disease and its treatment consequences.

### Behavioral coping strategies

Many participants in the present study stated that they tend to modify their lifestyle and diet to correct unhealthy lifestyles in the past or to prevent the recurrence of the disease. Qualitative studies of BC survivors in other countries have reported changes in nutritional habits and styles to maintain health and reduce fatigue caused by the BC. In most studies, using a low-fat diet, consuming more fruits and vegetables, and avoiding high consumption of red meat have been mentioned as beneficial changes in diet. The results of these studies are consistent with the results of the present study [41–43].

Another way to deal with BC was changing and correcting unhealthy lifestyle habits. As reported in other studies in Iran, various ways such as increasing physical activity, such as working at home, walking, going to gyms, and hiking, were mentioned. Similar results have been reported in other qualitative studies [44]. In a qualitative study on Chinese-Australian BC survivors, participants reported that exercise kept negative thoughts away from them. Also, the participants reported doing Tai chi (thought to be very useful in combatting depression), walking, and using weight machines at the gym to cope with their disease [41].

In a qualitative study of British cancer survivors, most participants knew that physical activity is good for general health. Some stated the benefits of physical activity relating to cancer and other chronic conditions, such as cardiovascular disease [45]*.*

Furthermore, according to a qualitative study on cancer survivors in Taiwan, most of the participants believed that cancer was a reflection of their past unhealthy lifestyles. To avoid problems, they tried to adjust their lifestyle, change their unhealthy eating habits, get more exercise, stop smoking and drinking, and deal with their stress [46].

Another coping style with BC in the present study was returning to routine activities and lifestyles. In other qualitative studies, BC survivors were willing to return to normal activities. They believed that activities related to home, job duties, self-care, child care, and participation in social activities lead to better coping with the disease and treatment complications. Also, the patients’ feelings of life satisfaction increased their quality of life and even improved their body image [21, 47, 48]. An important finding in Yamani Ardakani [49] on body image and its relationship with coping strategies in BC survivors was that employed, and educated women with BC had better body image than the others [49]. Also, cancer survivors in Taiwan improved their physical and emotional wellbeing and stated that participating in social activities resulted in greater satisfaction with life [46].

Another strategy used in the present study to cope with BC was specialized cancer support consultation services. In confirmation of the results of the present study, other qualitative studies also expressed the need for physical care and emotional counseling centers for cancer patients and people who have undergone hysterectomy [50–52]. However, for some patients or survivors, geographical access to such services was limited [48]. In line with our findings, other studies reported that positive support, especially from family, friends, and healthcare professionals, helped women with BC deal with the uncertainty they felt after the diagnosis [21]. In a mixed-method study about coping strategies during BC in Latina women, the participants expressed the need for an online community with doctors and program references in their areas and the ability to interact with other women who are going through a similar process or who have already gone through it [53]. A qualitative study on African American women BC survivors revealed that family is at the core of African American women’s strength. They cited their family as their most important source of support [54].

Another way to cope with BC was to search for more information about the disease, its course, complications, treatments, and medical and counseling services. Although one participant in the present study was reluctant to learn much about BC, other studies have shown that being aware of one's current condition increases cognitive and behavioral adaptation and leads to resistance against stress, redirects inappropriate coping activities, facilitates the problem-solving process, and results in the ability to tolerate increased levels of stress [48, 55]. So, it seems vital for health care providers to increase women’s self-awareness, considering the intense internalized fear of BC and its perceived incurability in women. It is also necessary that the mass media provide educational programs to focus on enhancing knowledge about BC and refining general beliefs transparently [56]. According to a qualitative study on Latina women, online resources may be a helpful way to disseminate knowledge and awareness to provide a unique support system [53]. The results of the present study and Smith et al. [45] study on British cancer survivors showed that limited information was given from oncology health professionals on how to achieve adequate levels of physical activity and many participants sought information via other methods, such as the media and websites [45]. Also, Khakbazan et al. [57] reported difficulty in accessing health care services for some reasons, such as distance and lack of knowledge of breast clinic locations and structural factors related to the health services, such as the long process of admission and challenges with referral systems were identified as factors that delayed timely medical help [57]. The results of observational studies and systematic reviews showed that women with BC use behavioral strategies such as seeking social support to cope with the disease [58–60].

### Emotional coping strategies

Many of the participants stated that in the early stages of diagnosis, they could not believe that BC had happened to them. In other studies, the experience of the denial phase in the early stages of diagnosis has been reported [44, 57, 61]. A study on the quality of life in Iranian BC survivors reported that when participants are faced with a BC diagnosis, they will experience various emotions such as fear, shock, disbelief, sadness, hostility, anger, depression, anxiety, and other feelings of psychosocial distress [62]. Khakbazan et al. [63] argue that emotional expressions such as denial, lack of concern, and optimism about the nature of symptoms could even lead to patient delays in seeking medical help for diagnosis.

In the present study, different methods of focusing on positive aspects of life were used to cope with BC. For example, they were wearing nice clothes, wearing wigs and special bras, not interacting with negative people, and thinking positively. Our findings have supported other qualitative studies [20, 48, 64–66]. For example, Yamani Ardakani [49] and Hajian [21], in qualitative studies on BC survivors, stated that the most important strategies for coping with BC were problem-focused coping strategies, acceptance, and positive and constructive thoughts [21, 49].

In this study, the patients believed their disease was a spiritual fate, a test conferred on them by God. They believed that the disease and its cure were in the hands of God and by the will of God, and in this way, they found peace. There has been a positive role of prayer and trust in God in the present study and other qualitative studies that have been done in different parts of Iran on women with BC [20, 62, 65–67]. In a qualitative study using the phenomenological approach to describe the ways Iranian women coped with BC-related complications and changes in their lives, most of the participants believed that they got cancer because it was God’s will; in some cases, the patients believed that “the disease came from God; we cannot prevent it, and it does not matter what we do.”[65]. A qualitative study of African American women BC survivors also showed that the participants believed that God enabled their survival of BC and worked every day of their lives [54]. Praying and reliance on a supernatural power have been reported in qualitative studies in other countries with different religions [15, 41, 68–70].

Another method of coping with BC in the present study was to rely on the help and support of family, relatives, and friends. The most common people who helped participants adapt during diagnoses and treatments and complications of illness and treatment were family members, especially spouses, mothers, neighbors, and friends. This has been confirmed in other qualitative studies in Iran on women who have had BC. Khakbazan et al. found that seeking social support and having supportive interactions with relatives had an influential role in the health-seeking behavior of BC patients [71]. In particular, the role of the spouse in adapting to women's diseases is critical and improves mental health and quality of life [20, 62, 65–67, 72, 73]. A meta-ethnographic synthesis on the help-seeking behaviors of women with BC revealed that family and other relatives tried to provide emotional and financial support for women, as well as to reassure, encourage, and advise women to seek treatment. In some cases, the pressure brought to bear by others (spouses, relatives, and colleagues) resulted in seeking medical help [57]. Studies in other countries have also reported the crucial role of the spouse in adapting to BC. A study about spousal support strategies for patients with BC in China showed that there was a role change for the husbands, from spouse to protector and caregiver, upon diagnosis and during treatment, and a return to the role of a spouse following the completion of treatment [53, 74].

The feelings of sympathy, compassion and some inappropriate behavior from friends, relatives, and the larger society posed a challenge for women coping with BC. A study on Korean women BC survivors revealed that some participants did not describe the role of some relatives, such as their sister or daughter-in-law, as supportive and friendly. They were forced to stay away from them because of their negative thoughts and words. Similar results have been reported in other studies [15]. Fasihi Harandi et al. [62] reported that most participants kept the illness as a personal issue because they did not like a negative and pitying reactions from family, friends, colleagues, and society [62].

In this study, we found that participation inhomogeneous groups was another way that affected the participants’ coping responses. A systematic review on peer support interventions for BC highlights peer support interventions for improving quality of life and reducing distress among BC patients [75]. Peer support helps the patients by providing hope and a way to cope, replacing other social roles weakened by disease, and offering understanding through shared experiences [14, 48, 76]. Establishing such groups seems essential as there is no organized group for BC survivors in Iran. According to some quantitative studies [60, 77] and a systematic review on coping strategies in BC patients [58], spiritual coping strategies play a significant role in the adjustment period. There was a positive and significant relationship between the study participants’ spiritual coping and cancer adjustment. BC patients also used other emotional strategies such as positive attitudes, hope, and optimism, accepting the disease, and deliberate denial [58]. Also, observational studies on BC survivors showed that the most commonly used problem-focused coping strategies included religious coping strategies and acceptance [69, 78].

One of the strengths of the present study is that it explores the emotional and behavioral coping strategies of BC survivors in Iran. While other studies that have been done in Iran, most of them have focused only on the aspect of psychological coping styles. Second, the present study was conducted in Mashhad, where patients are usually referred from other cities, and it was possible to select participants with maximum cultural diversity and different socio-economic levels.

The limitation of the present study was that it was conducted in the Iranian-Islamic community and can only be generalized to similar cultures. The present study was conducted in the east of Iran. Due to the cultural differences in different regions of Iran, the results of the present study for other regions of the country should be used with caution. Also, we explored coping strategies in primary and non-invasive BC survivors; therefore, the transferability of these results to the incurable setting or other cancer types is not desirable. Due to the qualitative nature of the study, the sample size was small, and this limited the transferability of the findings. However, because of the importance of conceptualization in qualitative studies, conceptual generalization (the richness, depth, and thickness of the data and diversity of the participants’ experiences) is more important than numerical generalization (the number of the participants). The other limitation of this study was that the analysis was not grounded in a specific behavioral framework, although an attempt was made to approach the data from a behavioral perspective as much as possible.

## Conclusion

Our findings provided an in-depth understanding of Iranian women’s strategies for coping with BC. The coping strategies adopted by Iranian BC survivors included emotional and behavioral strategies. Understanding the coping strategies of women diagnosed with BC could help health care providers to manage holistically the disease and provide physical, psychological, and social support. Even though health care systems provide services to improve the quality and quantity of patients’ lifestyles, these services are limited to the physical problems of patients, and their psychological issues are not taken into account, and this leads to severe psychological consequences for the survivors. It seems that a trained team of oncologists, psychiatrists, mental health professionals, and reproductive health specialists needs to contribute significantly to the upgraded coping of patients with cancer, which could lead to enhanced health promotion and a higher quality of life. Also, interventions to reduce cancer stigma should be developed. Additionally, providing services for quality social, emotional, and behavioral and special training services, including training in coping skills for BC survivors, should be considered in clinical oncology settings.

## Data Availability

The datasets used and/or analyzed during the current study are available from the corresponding author on reasonable request.
