# Correction to: How do I deal with breast cancer: a qualitative inquiry into the coping strategies of Iranian women survivors

**DOI:** 10.1186/s12905-022-01884-x

**Published:** 2022-07-25

**Authors:** E. Manouchehri, A. Taghipour, A. Ebadi, F. Homaei Shandiz, R. Latifnejad Roudsari

**Affiliations:** 1 Department of Midwifery, Faculty of Nursing and Midwifery, Mashhad Medical Sciences, Islamic Azad University, Mashhad, Islamic Republic of Iran; 2 Department of Midwifery, School of Nursing and Midwifery, Mashhad University of Medical Sciences, Mashhad, Islamic Republic of Iran; 3 Department of Epidemiology, School of Public Health, Mashhad University of Medical Sciences, Mashhad, Islamic Republic of Iran; 4 Social Determinants of Health Research Center, Mashhad University of Medical Sciences, Mashhad, Islamic Republic of Iran; 5 Behavioral Sciences Research Center, Lifestyle Institute, Baqiyatallah University of Medical Sciences, Tehran, Islamic Republic of Iran; 6 Nursing Faculty, Baqiyatallah University of Medical Sciences, Tehran, Islamic Republic of Iran; 7 Cancer Research Center, Mashhad University of Medical Sciences, Mashhad, Islamic Republic of Iran; 8 Nursing and Midwifery Care Research Center, Mashhad University of Medical Sciences, Mashhad, Islamic Republic of Iran

## Correction to: BMC Women’s Health (2022) 22:284 https://doi.org/10.1186/s12905-022-01865-0

Following the publication of the original article [1], the author name ‘R. Latifnejad Roudsari’ has been misspelled as ‘R. Latifnejad Roudsarii’.

The original article has been corrected.
